# Regulation of DDAH1 as a Potential Therapeutic Target for Treating Cardiovascular Diseases

**DOI:** 10.1155/2013/619207

**Published:** 2013-06-26

**Authors:** Xiaoyu Liu, John Fassett, Yidong Wei, Yingjie Chen

**Affiliations:** ^1^Shanghai Tenth People's Hospital, Tongji University, Shanghai, China; ^2^Cardiovascular Division and Lillehei Heart Institute, University of Minnesota, Minneapolis, MN 55455, USA

## Abstract

Asymmetric dimethylarginine (ADMA) is an endogenous nitric oxide synthase inhibitor that blocks nitric oxide production, while congestive heart failure is associated with increased plasma and tissue ADMA content. Increased plasma ADMA is a strong and independent predictor of all-cause mortality in the community and the strongest predictor of mortality in patients after myocardial infarction. Recent studies demonstrated that dimethylarginine dimethylaminohydrolase-1 (DDAH1) is the critical enzyme for ADMA degradation and thereby plays an important role in maintaining cardiovascular nitric oxide bioavailability. Interestingly, activation of the farnesoid X receptor (FXR) through the bile acid ursodeoxycholic acid (UDCA) or synthetic FXR agonists, such as GW4064, can increase DDAH1 expression. Thus, modulating DDAH1 activity through FXR receptor agonists such as UDCA could be a therapeutic target for treating reduced nitric oxide bioavailability in congestive heart failure and other cardiovascular diseases.

## 1. Introduction

Congestive heart failure (CHF) is a major cardiovascular disease of epidemic proportion that has increased in prevalence in the past few decades. Nitric oxide (NO) activates soluble guanylyl cyclase, and the resultant increase of cGMP and activation of cGMP-dependent protein kinases (PKG) regulate vasomotor tone, blood flow, angiogenesis, vascular endothelial cell growth/proliferation, and injury repair. NO is known to exert protective effects on the cardiovascular system. Impaired NO signaling is a hallmark of CHF and many other cardiovascular diseases such as hypertension, stroke, coronary disease, atherosclerosis, and diabetes. Thus, responses to agonists or shear stress that rely on NO to cause coronary or systemic vasodilatation are attenuated in CHF, indicating decreased NO bioavailability [1–4]. Reduced NO bioavailability causes hypertension [5], coronary disease, atherosclerosis and aging-dependent CHF in experimental animals [6], indicating an important role of NO in attenuating CHF and other cardiovascular diseases causing CHF.

**Figure 1:**
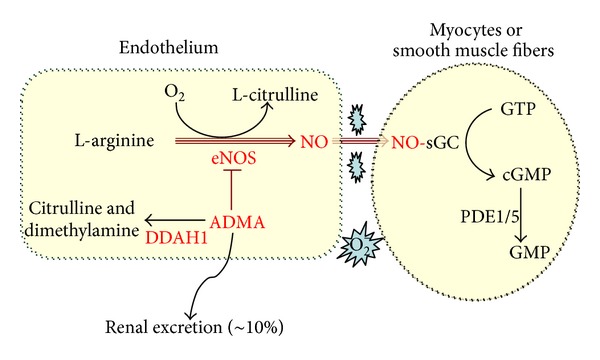
DDAH1 regulates NO production through degrading ADMA.

Asymmetric dimethylarginine (ADMA) is an endogenous NO synthase (NOS) inhibitor that blocks NO production and increases NOS-derived ROS generation. CHF is associated with increased ADMA levels in the heart and plasma. Increased plasma ADMA is a strong and independent predictor of all-cause mortality in the community [7] and the strongest predictor of mortality in patients with CHF [8]. Dimethylarginine dimethylaminohydrolase-1 (DDAH1) degrades ADMA and thereby enhances NO/cGMP signaling (Figure 1). Recent studies have demonstrated that DDAH1 is essential for ADMA degradation, indicating that DDAH1 plays an important role in maintaining cardiovascular NO bioavailability. Thus, elevated DDAH1 activity could be an important therapeutic target for increasing NO bioavailability in CHF and other cardiovascular diseases. 

### 1.1. Reduced NO Bioavailability Contributes to CHF Development

NO synthesis is catalyzed by a family of proteins, the NO synthases (NOS). At least three NOS isoforms exist in mammalian cells: endothelial NOS (eNOS), neuronal NOS (nNOS), and inducible NOS (iNOS). eNOS and nNOS are constitutively expressed (cNOS) in many cell types, and produce NO in response to increased cytosolic Ca^++^ (Ca^++^-dependent NOS). In the normal heart, eNOS is highly expressed in coronary endothelial cells and also moderately expressed on the sarcolemma of cardiac myocytes. Myocardial nNOS expression is low and mainly expressed on sarcoplasmic reticulum of cardiac myocytes, where it acts to regulate Ca^++^ dynamics. iNOS is expressed in response to inflammation or cytokine stimulation and can produce much larger quantities of NO for a sustained period of time in the absence of elevated Ca^++^ (Ca^++^-independent NOS).

Loss of NO bioavailability and cGMP production is a key feature of endothelial dysfunction in diseases such as hypertension and heart failure. One contribution to loss of NO-cGMP signaling is decreased NO production by NOS. Under conditions of oxidative stress or reduced substrate availability, NOS activity can become disrupted (NOS uncoupling; further described later), so that NOS produces super oxide, rather than NO [9]. In addition, elevated superoxide in cardiomyocytes or endothelial cells can interact with and scavenge NO before it can beneficially activate guanylate cyclase to produce cGMP. Furthermore, heart failure is associated with elevated expression of phosphodiesterase 5 (PDE5), which degrades cGMP and further reduces NO-cGMP dependent signaling. Thus NO-cGMP signaling is reduced through several mechanisms in the failing heart. Several studies using transgenic or knockout mouse models have now confirmed that NO-cGMP signaling significantly influences the development of myocyte hypertrophy and dysfunction during aging [6, 10, 11] and in response to myocardial injury or overload [12, 13]. Thus, transgenic mice overexpressing eNOS are protected from myocardial infarct-induced LV remodeling and the development of CHF [12]. Conversely, progressive cardiomyocyte hypertrophy, interstitial fibrosis, LV dilation, and dysfunction, that develops in the residual surviving tissue after myocardial infarction, are exacerbated in eNOS KO mice as compared with control wild-type mice [13]. Several laboratories have also reported that eNOS KO exacerbated LV dysfunction in response to left ventricular pressure overload (transverse aortic constriction; TAC) [14, 15]. Moreover, cardiomyocyte-restricted restoration of eNOS (overexpressing eNOS in eNOS KO mice) attenuates TAC-induced ventricular remodeling [16], suggesting a cardiomyocyte specific protective effect of eNOS expression under conditions of pressure overload. Cardiomyocyte specific eNOS overexpression in a wild-type background also attenuated TAC-induced LV remodeling [17]. These studies indicate that cardiomyocyte-restricted eNOS expression can protect the overloaded heart from ventricular dysfunction. These data demonstrated that the cNOS-derived NO-cGMP signaling pathway exerts cardioprotective effects against the development of LV hypertrophy and CHF. In contrast to the cardioprotective effect of eNOS, cardiac-specific iNOS expression can contribute to the development of CHF [18]. iNOS expression is increased in failing hearts [19, 20], and we [21] demonstrated that iNOS KO or selective pharmacologic iNOS inhibition with 1400 W protected the heart from TAC-induced LV hypertrophy and dysfunction. The finding that eNOS and iNOS have opposite influences on LV adaptation to pressure overload is explained by differences in the cell type and subcellular location of these NOS isoforms and by the fact that iNOS can produce much larger quantities of NO for a sustained period of time in the absence of elevated Ca^++^.

### 1.2. ADMA Accumulation Is Associated with CHF and Other Cardiovascular Diseases

The effect of endogenous NOS inhibitors on NO bioavailability has been an area of intense research in recent years. There are two compounds that can inhibit NOS, N-monomethyl-arginine (NMMA), and ADMA, which both reduce NO synthesis by competing with arginine for NOS binding [22]. Plasma NMMA concentrations are much lower compared with plasma ADMA concentrations. NMMA is formed when protein-incorporated arginine is methylated by the enzymes protein arginine methyltransferases- (PRMT-) 1 or PRMT-2. PRMT-1 can subsequently methylate NMMA, resulting in the formation of ADMA, whereas PRMT-2 can methylate NMMA into symmetric dimethylarginine (SDMA). After proteolysis, the methylated arginines are released as unbound forms in the cytosol where NMMA and ADMA are able to inhibit NOS. SDMA is not able to inhibit NOS. Because only small amounts of NMMA are found in the plasma, ADMA is considered the major endogenous NOS inhibitor. ADMA and L-NMMA are eliminated principally by DDAH with a small contribution from renal excretion. 

Plasma ADMA levels are elevated in CHF [23, 24], hypertension [25], diabetes [26], and atherosclerosis [27, 28], and increased ADMA is believed to contribute to endothelial dysfunction in these conditions. In agreement with ADMA accumulation in CHF patients, we demonstrated that rapid ventricular pacing-induced CHF in dogs was associated with increased plasma ADMA that was accompanied by the development of progressive coronary endothelial dysfunction [29]. In addition, administration of ADMA attenuated acetylcholine induced coronary vessel dilation in normal animals [29]. Several investigators have reported that increased plasma ADMA levels are associated with an increased risk for developing angina pectoris, myocardial infarction, or cardiac death [7]. However, despite the association between increased levels of ADMA and CHF or other cardiovascular diseases, it is uncertain whether chronic ADMA accumulation can actually cause or exacerbate the development of myocardial dysfunction or CHF. 

### 1.3. ADMA Enhances NOS-Derived O_**2**_^−^ and Peroxynitrite (ONOO^−^)

Although the most obvious consequence of increased levels of ADMA and L-NMMA is to inhibit NO production, recent reports indicate that the endogenous NOS inhibitors may cause NOS to generate O_2_^−^ rather than NO. Normally, NOS transfers electrons from NADPH, via the flavins FAD and FMN in the carboxy-terminal reductase domain, to the heme in the amino-terminal oxygenase domain, where the substrate L-arginine is oxidized to L-citrulline and NO. The flow of electrons within NOS is normally tightly regulated but, if disturbed, oxygen reduction and NO generation can become uncoupled so that O_2_^−^ is generated by the oxygenase domain. This uncoupling can occur when NOS is exposed to oxidant stress (including peroxynitrite), when it is deficient of the reducing cofactor BH4 [30, 31] or when it is deprived of its substrate L-arginine [32, 33]. BH4 is required for iNOS dimerisation [34, 35] and stabilizes the dimeric forms of eNOS, nNOS, and iNOS once formed [34]. Thus, BH4 depletion (or oxidation of BH4 to BH2) can induce NOS-derived O_2_^−^ generation [9, 30, 34, 35]. Deprivation of the substrate l-arginine can also induce NOS to generate O_2_^−^ and ONOO^−^ [32, 33, 36]. We recently found that both iNOS and eNOS monomer were increased in failing hearts from wild-type mice in response to TAC, and this was associated with increased myocardial superoxide production [21]. We found that iNOS KO or the selective iNOS inhibitor 1400 W protected the heart against TAC-induced LV dysfunction and oxidative stress [21]. However, it is not fully clear whether iNOS-derived ROS is due to substrate deficiency. In a manner similar to substrate deficiency, several *in vitro* studies have demonstrated that the addition of ADMA or L-NMMA,which act as a competitive inhibitors of l-arginine, caused O_2_^−^ generation by purified NOS protein [22, 37], in cultured human endothelial cells [38, 39], isolated arterioles from rat gracilis muscle [40], and in a murine lung epithelial cell line LA-4 [41]. *In vitro* studies have demonstrated that the NOS inhibitor N-monomethyl-L-arginine (L-NMMA) is also capable of inducing NOS uncoupling through multiple mechanisms such as heme loss [37]. Importantly, administration of tetrahydrobiopterin, which prevents NOS uncoupling, can significantly attenuate ROS production, pressure overload-induced cardiac hypertrophy, and heart failure, indicating that the loss of NO production, as well as the increased ROS production that results from NOS uncoupling, is important in the development of heart failure. 

Thus, ADMA inhibition of NO production, and possibly ADMA-induced NOS uncoupling and production of superoxide, may act as a double-edged sword in endothelial and cardiomyocyte pathophysiology. Identification and understanding of the mechanisms that reduce ADMA accumulation are therefore, clinically important. 

### 1.4. DDAH1 Is Essential for ADMA Degradation

DDAH1 was originally identified by Ogawa et al. in 1987 [42]. DDAH2 was identified in 1999 [43]. Previous existing concepts regarding tissue or cell-specific DDAH1/2 biology and their function in regulating NO production in various tissues are based on the reports that DDAH1 and DDAH2 have comparable activities for degrading ADMA and L-NMMA [43], as well as the report that DDAH1 is minimally expressed in the heart [43, 44], vessels [43], and vascular endothelial cells [45]. Accordingly, it was originally accepted that DDAH2 plays the major role in regulating ADMA and L-NMMA levels in the heart and vessels, while DDAH1 plays the major role in degrading ADMA and L-NMMA in neuronal tissues. However, recent studies clearly demonstrated that DDAH1 is important in regulating systemic ADMA and L-NMMA and cardiovascular NO bioavailability [46]. Thus, using endothelial specific DDAH1 gene deficient mice (endo-DDAH1 KO), we found that endo-DDAH1 KO caused significant decreases of DDAH1 in vascular tissues, increased tissue and plasma ADMA, reduced acetylcholine-induced NO production and vessel dilatation, and resulted in systemic hypertension [46]. Consistent with the above findings, studies from Cooke and Associates demonstrated that a moderate 2-3-fold overexpression of DDAH1 in transgenic mice was sufficient to significantly decrease plasma ADMA and to cause a moderate decrease of aortic pressure [47]. These findings imply that DDAH1 in vascular endothelial cells plays an important role in degrading the NOS inhibitors and regulating vascular tone. The increased systemic hypertension observed in DDAH1 KO mice in which ADMA levels were increased and NO production was decreased suggests that DDAH1 degradation of ADMA is physiologically important and may help protect against cardiovascular diseases in which ADMA levels are elevated.

Most importantly, we demonstrated that DDAH activity was totally abolished in all tissues obtained from global DDAH1 deficient mice while the expression of DDAH2 was unaffected in these tissues [48]. In other words, tissues obtained from our global DDAH1 KO mice are unable to degrade ADMA or NMMA [48]. Consistent with our findings, Dr. Leiper et al. also demonstrated that DDAH activity was reduced ~50% in tissues obtained from heterozygous DDAH1 KO mice [49]. Furthermore, we found that selective gene silencing of DDAH1, but not DDAH2, caused accumulation of ADMA and decreased NO production in cultured endothelial cells [46], while overexpression of DDAH1 (but not DDAH2) decreased ADMA content in cultured endothelial cells [50]. These findings clearly indicate that DDAH1 is the critical enzyme for ADMA and L-NMMA degradation, while DDAH2 has no detectable role in ADMA degradation. Technical limitation and inappropriate experimental methods used for detecting ADMA degradation are likely the culprit in defining DDAH2 as an enzyme for ADMA degradation. Because DDAH activity is reduced in the failing heart [29] and this likely contributes to increased ADMA levels and endothelial dysfunction, identifying new mechanisms to increase DDAH1 expression or activity may be clinically relevant in the treatment of heart failure.

### 1.5. The Potential Role of Ursodeoxycholic Acid (UDCA) in Vascular NO Bioavailability and Blood Flow

Bile acid ursodeoxycholic acid (UDCA) is a major component of bear bile, which has been used extensively in traditional Chinese medicine. UDCA has been on the market in Japan since the 1950s and in Western countries since the mid-1980s [51]. UDCA or bile acids contribute to several essential functions, including cholesterol catabolism and intestinal lipid emulsification. In addition to their role as detergents, bile acids can also act as endocrine signaling molecules via activation of nuclear receptors, including farnesoid X receptor (FXR) and pregnane X receptor [52] to achieve profound effects on hepatic lipid and glucose metabolism [53]. FXR is activated by compounds such as UDCA, chenodeoxycholic acid, and cholic acid [54, 55] and by the synthetic compound GW4064 [56]. FXR plays an important role in maintaining cholesterol, triglyceride, and glucose homeostasis [56–58]. Activation of FXR with bile acids or GW4064, or hepatic expression of constitutively active FXR, significantly lowers plasma triglyceride, cholesterol, and glucose levels [56, 57]. Conversely, FXR gene deletion increased plasma cholesterol and triglyceride levels [58]. UDCA is currently employed in the clinical treatment of diverse hepatobiliary disorders, including primary biliary cirrhosis [59] and other liver diseases. Interestingly, genetic disruption of eNOS or nNOS has been shown to alter lipid metabolism, resulting in increased fat deposition in the liver [60, 61]. It would be interesting to find out whether FXR-mediated increase of DDAH1 activity influences lipid metabolism through preservation of NOS activity. UDCA can also attenuate ER stress [62], a phenomena often observed in CHF. One study demonstrated that 6-week UDCA therapy improved endothelium-dependent vasodilatation and arterial blood flow in patients with HF under conditions of impaired nitric oxide production [55]. In a double-blind, randomized, and placebo-controlled clinical trial, von Haehling et al. demonstrated that UDCA significantly improved peak postischemic blood flow in the arm and that there was a trend towards improved peak postischemic blood flow in the leg, while liver function was also improved [63]. Most interestingly, a recent report demonstrated that DDAH1 is a downstream target gene of FXR [64]. Thus, activation of FXR with GW4064 dose dependently increased DDAH1 gene transcription in the liver through a FXR response element, and this was associated with a decrease of plasma ADMA [64]. A separate study by a different group also recently reported that activation of FXR with GW4064 increased DDAH1 gene expression in the liver and kidney and decreased plasma ADMA [65]. Activation of FXR with bile acids was also found to enhance tumor angiogenesis [58]. Interestingly, FXR is expressed in cardiomyocytes [66] and endothelial cells [67], but whether activation of FXR is able to increase DDAH1 gene expression in the cardiovascular system, or can attenuate pressure overload-induced ventricular dysfunction, has not been studied. The effects of UDCA and/or other bile acids in attenuating plasma cholesterol and triglyceride levels [58] and potential for increasing vascular NO bioavailability through modulating DDAH1 expression suggest that UDCA (and/or other bile acids) may exert cardiac protective effect in the failing heart. 

Collectively, the current scientific literature in the field indicates that ADMA attenuates vascular NO bioavailability in the cardiovascular system, that DDAH1 plays the major role in ADMA degradation, and that activation of FXR increases DDAH1 expression. Together these findings suggest that increasing DDAH1 expression through FXR activation could be an important therapeutic target for treating reduced NO bioavailability in CHF and other cardiovascular diseases.
